# Hybrid Code Index Modulation Based on Multi-Carrier Differential Chaos Shift Keying

**DOI:** 10.3390/e28060579

**Published:** 2026-05-22

**Authors:** Xibei Yu, Chunyan Song

**Affiliations:** College of Computer and Control Engineering, Northeast Forestry University, Harbin 150040, China; 17377865057@163.com

**Keywords:** differential chaos shift keying, hybrid code index modulation, multi-carrier modulation, bit error rate, Walsh codes

## Abstract

A hybrid code index modulation based on multi-carrier differential chaos shift keying (DCSK), referred to as HCIM MC-DCSK, is proposed in this paper. In the proposed system, multiple data-bearing information signals are transmitted simultaneously with one reference signal. The number of separate physical channels required for data transmission is M + 1, where M is the number of subcarriers. These data-bearing information signals are separated by different Walsh codes. The chaotic signal and its Hilbert transform are utilized to complete the hybrid index modulation. In addition, analytical bit-error-rate expressions are derived for the proposed HCIM MC-DCSK system operating over AWGN and multipath Rayleigh fading channels. The spectral efficiency and data rate of the proposed system are analyzed. The validity of the analytical results and the superiority of the proposed system are confirmed through relevant simulations.

## 1. Introduction

Chaotic signals have many properties, such as noise-like characteristics, wide bandwidth, and extreme sensitivity to initial conditions, which make them widely utilized in the field of communications [1,2,3,4]. In recent years, chaotic modulations have garnered considerable attention in the field of wireless communications due to their strong anti-fading capability, low probability of interception, and high spectrum utilization [5,6].

Among various chaotic modulation schemes, differential chaos shift keying (DCSK) has been extensively studied over the past two decades as a relatively classic method [7]. The primary motivations for adopting DCSK in practical communication systems are threefold. First, DCSK is a non-coherent scheme that eliminates the need for chaotic synchronization at the receiver, which is notoriously difficult to achieve in dynamic multipath fading environments [8,9]. Second, DCSK exploits the excellent correlation properties of chaotic signals to provide inherent robustness against multipath interference without requiring complex channel estimation or equalization [10,11]. Third, the noise-like and aperiodic nature of chaotic carriers offers a low-probability-of-interception (LPI) characteristic, providing physical layer security advantages over conventional sinusoidal modulation schemes [12,13]. These features make DCSK particularly attractive for low-complexity IoT devices and short-range wireless applications.

However, DCSK has some significant limitations, such as a relatively high bit error rate (BER), low energy efficiency, and the use of delay components. To overcome these shortcomings, many improved DCSK schemes have been proposed, such as frequency-modulated differential-chaos-shift-keying communication (FM-DCSK) [14], code-shifted DCSK (CS-DCSK) [15], short-reference DCSK (SR-DCSK) [16], and so on. To achieve high data rate, the multi-carrier (MC) scheme was adopted in chaos-based communication systems, such as multi-carrier DCSK (MC-DCSK) [17], multi-carrier M-Ary differential chaos shift keying (MC-MDCSK) [18], multi-carrier chaos shift keying (MC-CSK) [19], and so on.

Index modulation (IM) has recently been introduced into chaos-based communication systems, primarily to elevate the data rate and enhance energy efficiency [20]. An efficient and reliable communication scheme can be achieved through the combination of IM and the basic chaotic modulation scheme [21]. Inspired by IM and MC-DCSK, [22] proposed a carrier indexed DCSK (CI-DCSK) scheme where the index of the active subcarrier carries additional data bits, aiming to achieve low power consumption and improved energy efficiency. By combining DCSK and code index modulation (CIM), the authors of [23] proposed a code index modulation DCSK (CIM-DCSK) scheme that outperforms the traditional DCSK system over frequency selective fading channels. For realizing high energy efficiency, high spectral efficiency and high data rate, a multi-carrier differential chaos shift keying communication system with hybrid index modulation (HIM-MC-DCSK) was proposed in [24]. A novel differential chaotic time-shift keying system (CTIM-DCSK) was also proposed which utilizes frequency and time resources to achieve high-data-rate transmission [25]. Moreover, to meet the demand for rapidly growing data traffic, a high-data-rate chaotic communication scheme was proposed, namely, the Hybrid Carrier Coding and Reference Index Modulation Differential Chaotic Shift Keying system (HCCRIM-TF-DCSK) [26]. However, the improvements in data rate and energy efficiency offered by the above solutions are limited.

To further enhance data rate and energy efficiency, in this study, a novel hybrid index modulation and code index modulation based on multi-carrier differential chaos shift keying communication system (HCIM MC-DCSK) is proposed based on HIM-MC-DCSK and CIM-DCSK schemes. In this system, all subcarriers can be utilized to transmit information bits, thereby further enhancing the transmission data rate and avoiding waste of spectrum resources. In short, the main contributions are as follows:

(1) A new chaos communication system, HCIM MC-DCSK, is proposed. In this system, hybrid index modulation—code index modulation and multi-carrier modulation—is utilized. Different Walsh codes are employed to separate multiple carrying data.

(2) The bit-error-rate (BER) expression for the HCIM MC-DCSK system is derived for both additive white Gaussian noise (AWGN) channels and multipath Rayleigh fading channels. Simulation results validate the effectiveness of the theoretical analysis. Through comparative analysis of the proposed system, the CI-DCSK system, the PT-CIM-DCSK system, the CSK system, the QCSK system and the QCAM system, the advantages of the proposed system are demonstrated.

(3) This paper analyzes the spectral efficiency (SE) and data rate of the proposed system, comparing it with other systems. The proposed system significantly outperforms competing solutions in terms of spectral efficiency and data rate.

The remainder of this paper is structured as follows. Section 2 presents the system model of the proposed system. Section 3 analyzes the performance of the proposed system. Section 4 presents simulation results and discussion. Section 5 provides conclusions.

## 2. System Model of HCIM MC-DCSK System

### 2.1. Transmitter

**Figure 1:**
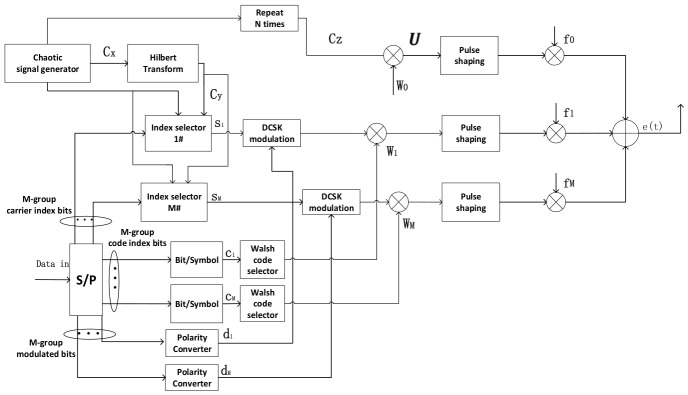
The block diagram of the HCIM MC-DCSK transmitter.

The structure of the HCIM MC-DCSK transmitter is given in Figure 1. The serial data sequence in Figure 1 is first converted into M parallel groups via a serial-to-parallel converter. Each information group includes three parts: N carrier index bits, N code index bits and N modulated bits. M groups of information signals are transmitted in parallel by using M orthogonal Walsh codes. In this system, M + 1 subcarriers are used. One subcarrier is utilized for transmitting the reference signal. And the remaining M subcarriers are used for transmitting information signals.

The chaotic signal $C_{x}=\{c_{x,u}\}(u=1,\;2,\ldots ,\theta )$ is generated by utilizing a second-order Chebyshev polynomial function (i.e., $x_{i+1}=1-2x_{i}^{2}$) with initial conditions $c_{1}=0.1184$. The length of the chaotic signal is θ. The Hilbert transformer is utilized for obtaining the orthogonal signal ${C_{y}=\{c}_{y,u}\}\;(u=1,\;2,\ldots ,\theta )$. The chaotic signal $C_{x}$ and its orthogonal signal $C_{y}$ satisfy $\sum_{u=1}^{\theta }c_{x,u}c_{y,u}=0$. The chaotic signal $C_{x}$ is subsequently repeated N times to expand into the chaotic repeated sequence $C_{z}$. The length of the chaotic repeated sequence is β, where β=Nθ. Then, the chaotic repeated sequence is multiplied with the Walsh code $W_{0}$ to obtain U, where $U=C_{z}⨂W_{0}$. Subsequently, U is input to the pulse shaping filter to form a chaotic reference signal $C_{R}\left(t\right)$ during the current symbol duration, where

(1) $$C_{R}\left(t\right)=\sum_{k=1}^{P_{2}}U_{k}h\left(t-kT_{c}\right)$$

In Equation (1), h(t) is the impulse response of the pulse shaping filter with a normalized energy, and $T_{c}$ is the chip time. The length of the Walsh code is $P_{1}$, and $P_{2}=P_{1}\cdot \beta$.

In Figure 1, M index selectors are adopted for realizing hybrid index modulation of the carrier index bits. If the carrier index bit satisfies $s_{m,n}=1$, the chaotic signal ${\{c}_{x,u}\}$ will be selected, and if the carrier index bit satisfies $s_{m,n}=0$, the orthogonal chaotic signal $\left\{c_{y,u}\right\}$ will be selected. For detailed specifications, refer to [24]. The output of the $m^{th}$ index selector is defined as:

(2) $$s_{m}=\left\{\begin{array}{c} \sum_{u=1}^{\theta }c_{x,u},\;s_{m,n}=1\;\\ \sum_{u=1}^{\theta }c_{y,u},\;s_{m,n}=0\; \end{array}\right.\;1\leq n\leq N,\;1\leq m\leq M$$

In Figure 1, the M groups of code index bits are respectively fed into bit-to-symbol converters to obtain the code index modulating symbol $c_{m}\in \left\{1,\ldots ,P_{1}\right\}$. The code index modulating symbol is utilized for selecting the corresponding Walsh code sequence. And the length of the Walsh code $P_{1}$ satisfies $P_{1}=2^{N}$ and $M<P_{1}$.

In Figure 1, the M groups of modulated bits are input into polarity converters to obtain the polarity symbols. Then the polarity symbols are fed into the DCSK modulations. If $Q_{m,n}=1$, the result of the polarity converter is $d_{m,n}=1$. If $Q_{m,n}=0$, $d_{m,n}=-1$. The outputs of the DCSK modulation are respectively multiplied with the Walsh code $W_{1},\ldots ,W_{M}$, which is selected from the Walsh code selector. Later, these products are put into the pulse shaping filters and then multiplied with M subcarriers $f_{1},\ldots f_{M}$, respectively. Simultaneously, the chaotic reference signal $C_{R}\left(t\right)$ is modulated onto the reference subcarrier $f_{0}$. Thus, the signal transmitted by the sender of the HCIM MC-DCSK system can be described as:

(3) $$e\left(t\right)=C_{R}\left(t\right)\cos\left(2\pi f_{0}+\varphi_{0}\right)+\sum_{m=1}^{M}e_{m}(t)\cos\left(2\pi f_{m}+\varphi_{m}\right)\;$$

where $e_{m}\left(t\right)=\sum_{k=1}^{P_{2}}V_{k}h\left(t-kT_{c}\right)=\sum_{k=1}^{P_{2}}(d_{m}s_{m}⨂W_{m})h\left(t-kT_{c}\right)$. $\varphi_{0}$ and $\varphi_{m}$ are respectively the phase angle of the reference subcarrier and the $m^{th}$ subcarrier. $d_{m}=\left\{d_{m,n}\right\},d_{m,n}\in \left\{-1,1\right\}$, 1≤n≤N.

### 2.2. Receiver

**Figure 2:**
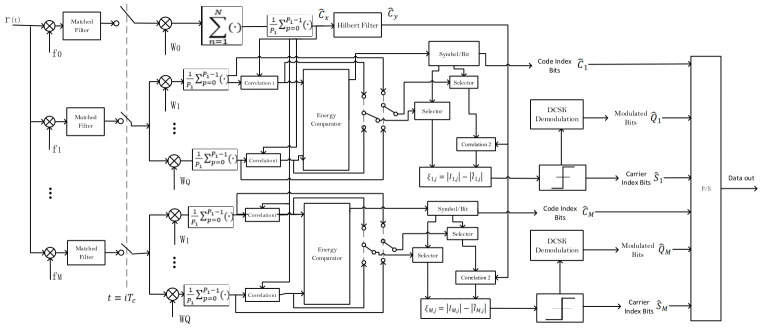
The block diagram of the HCIM MC-DCSK receiver.

The structure of the HCIM MC-DCSK receiver is given in Figure 2. The received signal through the channel can be represented as

(4) $$r\left(t\right)=\sum_{l=1}^{L}\lambda_{l}e\left(t-\tau_{l}\right)+n\left(t\right)$$

where L denotes the number of paths, and $\lambda_{l}$ and $\tau_{l}$ represent the channel coefficient and corresponding path delay, respectively. $n\left(t\right)$ denotes the additive white Gaussian noise (AWGN) with zero mean and variance N02. The received signal $r\left(t\right)$ is separated by multiplying with M + 1 synchronized local carriers at the subcarrier frequencies and then passed through M + 1 matched filters, respectively. Then, the outputs of the matched filters are sampled at every $iT_{c}$ time instant.

The averaged reference signal received can be expressed as

(5) $${\hat{c}}_{x,j}=\frac{1}{P_{1}}\sum_{n=1}^{N}\sum_{p=0}^{P_{1}-1}W_{0,p+1}\left[\sum_{l=1}^{L}\lambda_{l}W_{0,p+1}c_{z,p\theta +j-\tau_{l}}+n_{p\theta +j}\right]=\sum_{l=1}^{L}\lambda_{l}c_{x,j-\tau_{l}}+n_{R,j}$$

where $n_{R,j}=\frac{1}{P_{1}}\sum_{n=1}^{N}\sum_{p=0}^{P_{1}-1}W_{0,p+1}\cdot n_{p\theta +j}$.

The $m^{th}$ received information signal can be expressed as

(6) $$r_{q,j}=\frac{1}{P_{1}}\sum_{p=0}^{P_{1}-1}W_{q,p+1}\left[\sum_{l=1}^{L}\lambda_{l}d_{m,j-\tau_{l}}s_{m,j-\tau_{l}}W_{m,p+1}+n_{p\theta +j}\right]\;=\left\{\begin{array}{c} \sum_{l=1}^{L}\lambda_{l}d_{m,j-\tau_{l}}s_{m,j-\tau_{l}}+n_{q,j},\;q=m\\ n_{q,j},\;q\neq m \end{array}\right.$$

where $n_{q,j}=\frac{1}{P_{1}}\sum_{p=0}^{P_{1}-1}W_{q,p+1}\cdot n_{p\theta +j}$.

When q=m, the results after correlation 1 are defined as

(7) $$I_{m,j}=(\sum_{k=1}^{\theta }\sum_{l=1}^{L}\lambda_{l}d_{j,k-\tau_{l}}s_{j,k-\tau_{l}}+n_{m,k})\cdot (\sum_{k=1}^{\theta }\sum_{l=1}^{L}\lambda_{l}c_{x,k-\tau_{l}}+n_{R,k})$$

In energy comparator, the maximum value of the absolute value of (7) can be compared to recover the code index bits ${\hat{C}}_{m}$.

The Hilbert transform of the averaged reference signal ${\hat{C}}_{y}$ is obtained by the Hilbert filter, which is utilized for completing the correlation 2. The results of two correlations are directly subtracted to obtain a new decision metric $\xi_{q,j}$ as

(8) $$\xi_{q,j}=\left|I_{q,j}\right|-\left|{\tilde{I}}_{q,j}\right|\;j=1,\ldots ,N$$

Then, this new decision metric is sent to the threshold decision to recover the carrier index bit, namely,

(9) $${\hat{s}}_{m,j}=\left\{\begin{array}{c} 1,\;\xi_{q,j}>0\;\\ 0,\;otherwise \end{array}\right.\;j=1,\ldots ,N$$

The modulated bit ${\hat{d}}_{m,j}$ is estimated by

(10) $${\hat{d}}_{m,j}=\left\{\begin{array}{c} sgn\left(I_{q,j}\right),\;\left|I_{q,j}\right|>\left|{\tilde{I}}_{q,j}\right|\;\\ sgn\left({\tilde{I}}_{q,j}\right),\;\left|{\tilde{I}}_{q,j}\right|>\left|I_{q,j}\right| \end{array}\right.$$

## 3. Performance Analysis

### 3.1. BER

The maximum multipath delay is assumed to be much smaller than the symbol duration, i.e., $0<\tau_{lmax}\ll \beta$, thereby rendering inter-symbol interference (ISI) negligible [27,28]. Furthermore, the channel is assumed to be slowly fading with constant coefficients over each symbol period.

#### 3.1.1. Formulation of System BER

In every HCIM MC-DCSK transmission, $m_{c}+m_{s}+m_{q}$ bits are sent, where the $m_{c}$ bits are used as the code index bits, the $m_{s}$ bits are used as the carrier index bits, and the $m_{q}$ bits are used as the modulated bits. The BER of the code index bits is denoted as $P_{CIM}$. The BER of the carrier index bits is denoted as $P_{INDEX}$. The BER of the modulated bits is denoted as $P_{MOD}$. Thus, the system BER $P_{system}$ can be given as

(11) $$P_{system}=\frac{m_{c}}{m_{c}+m_{s}+m_{q}}P_{CIM}+\frac{m_{s}}{m_{c}+m_{s}+m_{q}}P_{INDEX}+\frac{m_{q}}{m_{c}+m_{s}+m_{q}}P_{MOD}$$

#### 3.1.2. Derivation of $P_{eds}$

Based on Equation (7), considering q=m, the output of correlation 1 can be expressed as

(12) $$I_{m,j}=(\sum_{k=1}^{\theta }\sum_{l=1}^{L}\lambda_{l}d_{j,k-\tau_{l}}s_{j,k-\tau_{l}}+n_{m,k})\cdot (\sum_{k=1}^{\theta }\sum_{l=1}^{L}\lambda_{l}c_{x,k-\tau_{l}}+n_{R,k})\;=\sum_{k=1}^{\theta }\sum_{l=1}^{L}\lambda_{l}^{2}d_{j,k-\tau_{l}}s_{j,k-\tau_{l}}c_{x,k-\tau_{l}}+\sum_{k=1}^{\theta }\sum_{l=1}^{L}\lambda_{l}d_{j,k-\tau_{l}}s_{j,k-\tau_{l}}n_{R,k}+\sum_{k=1}^{\theta }\sum_{l=1}^{L}\lambda_{l}c_{x,k-\tau_{l}}n_{m,k}+\sum_{k=1}^{\theta }n_{m,k}n_{R,k}$$

where

(13) $$A=\sum_{k=1}^{\theta }\sum_{l=1}^{L}\lambda_{l}d_{j,k-\tau_{l}}s_{j,k-\tau_{l}}n_{R,k}+\sum_{k=1}^{\theta }\sum_{l=1}^{L}\lambda_{l}c_{x,k-\tau_{l}}n_{m,k}\;$$

(14) $$B=\sum_{k=1}^{\theta }n_{m,k}n_{R,k}$$

And the output of correlation 2 can be expressed as

(15) $$\begin{array}{c} {\tilde{I}}_{m,j}=(\sum_{k=1}^{\theta }\sum_{l=1}^{L}\lambda_{l}d_{j,k-\tau_{l}}s_{j,k-\tau_{l}}+n_{m,k})\cdot (\sum_{k=1}^{\theta }\sum_{l=1}^{L}\lambda_{l}c_{y,k-\tau_{l}}+{\hat{n}}_{R,k})\\ =\sum_{k=1}^{\theta }\sum_{l=1}^{L}\lambda_{l}^{2}d_{j,k-\tau_{l}}s_{j,k-\tau_{l}}c_{y,j-\tau_{l}}+\sum_{k=1}^{\theta }\sum_{l=1}^{L}\lambda_{l}d_{j,k-\tau_{l}}s_{j,k-\tau_{l}}{\hat{n}}_{R,k}+\sum_{k=1}^{\theta }\sum_{l=1}^{L}\lambda_{l}c_{y,j-\tau_{l}}n_{m,k}+\sum_{k=1}^{\theta }n_{m,k}{\hat{n}}_{R,k}\; \end{array}$$

where

(16) $$C=\sum_{k=1}^{\theta }\sum_{l=1}^{L}\lambda_{l}d_{j,k-\tau_{l}}s_{j,k-\tau_{l}}{\hat{n}}_{R,k}+\sum_{k=1}^{\theta }\sum_{l=1}^{L}\lambda_{l}c_{y,j-\tau_{l}}n_{m,k}\;\;$$

(17) $$D=\sum_{k=1}^{\theta }n_{m,k}{\hat{n}}_{R,k}$$

We set $s_{m}=\sum_{u=1}^{\theta }c_{x,u}$, and $d_{j}=+1$. The mean of $I_{m,j}$ can be obtained as

(18) $$\mu_{1}=\mu_{I_{m,j}}=E\left[\sum_{k=1}^{\theta }\sum_{l=1}^{L}\lambda_{l}^{2}d_{j,k-\tau_{l}}c_{x,j-\tau_{l}}^{2}\right]+E\left[A\right]+E\left[B\right]=\sum_{l=1}^{L}\lambda_{l}^{2}\cdot E\left[\sum_{k=1}^{\theta }c_{x,j-\tau_{l}}^{2}\right]$$

where

(19) $$\sum_{k=1}^{\theta }c_{x,j-\tau_{l}}^{2}=\frac{E_{b}\cdot 3NM}{(M+1)P_{1}}$$

where $E_{b}$ is the energy of the transmitted bit. N denotes the number of bits for each dimension: carrier index, code index, and modulated data (3N bits per symbol in total).

Thus, the mean of $I_{m,j}$ can be expressed as

$$\mu_{1}=\frac{E_{b}\cdot 3NM}{(M+1)P_{1}}\sum_{l=1}^{L}\lambda_{l}^{2}$$

Similarly, the mean of ${\tilde{I}}_{m,j}$ can be obtained as

(20) $$\mu_{2}=\mu_{{\tilde{I}}_{m,j}}=0$$

The variance of $I_{m,j}$ and ${\tilde{I}}_{m,j}$ can be calculated as

(21) $$\sigma_{1}^{2}=\sigma_{I_{m,j}}^{2}=E\left[A^{2}\right]+E\left[B^{2}\right]=\frac{N_{0}}{P_{1}}\cdot \frac{E_{b}\cdot 3NM}{\left(M+1\right)P_{1}}\sum_{l=1}^{L}\lambda_{l}^{2}+\frac{N_{0}^{2}}{2{P_{1}}^{2}}$$

(22) $$\sigma_{2}^{2}=\sigma_{{\tilde{I}}_{m,j}}^{2}=E\left[C^{2}\right]+E\left[D^{2}\right]=\frac{N_{0}}{P_{1}}\cdot \frac{E_{b}\cdot 3NM}{\left(M+1\right)P_{1}}\sum_{l=1}^{L}\lambda_{l}^{2}+\frac{N_{0}^{2}}{2{P_{1}}^{2}}$$

Because $I_{m,j}$ and ${\tilde{I}}_{m,j}$ obey the Gaussian distribution [24,29], $\left|I_{m,j}\right|$ and $\left|{\tilde{I}}_{m,j}\right|$ obey the folded Gaussian distribution [30,31]. The mean and the variance can be respectively expressed as

(23) $$\mu_{f}=\sqrt{\frac{2\sigma^{2}}{\pi }}e^{-\frac{\mu^{2}}{2\sigma^{2}}}-\mu \cdot \mathrm{erf}\left(-\sqrt{\frac{\mu^{2}}{2\sigma^{2}}}\right)$$

(24) $$\sigma_{f}^{2}=\mu^{2}+\sigma^{2}-\mu_{f}^{2}$$

Thus, the mean of $\left|I_{m,j}\right|$ is obtained as

(25) $$\mu_{\left|I_{m,j}\right|}=\sqrt{\frac{2\sigma_{1}^{2}}{\pi }}e^{-\frac{\mu_{1}^{2}}{2\sigma_{1}^{2}}}-\mu_{1}\cdot \mathrm{erf}\left(-\sqrt{\frac{\mu_{1}^{2}}{2\sigma_{1}^{2}}}\right)=\Omega_{0}\cdot \sqrt{\sum_{l=1}^{L}\lambda_{l}^{2}\cdot E_{b}N_{0}}$$

where

(26) $$\Omega_{0}=\sqrt{\frac{2[\frac{3NM}{(M+1){P_{1}}^{2}}+\frac{1}{2\gamma_{b}{P_{1}}^{2}}]}{\pi }}e^{-\frac{1}{2\left[\frac{1}{\frac{3NM}{(M+1)P_{1}}\cdot \gamma_{b}}+\frac{1}{4{\left(\frac{3NM}{(M+1)P_{1}}\right)}^{2}{\gamma_{b}}^{2}}\right]}}-\frac{3NM}{\left(M+1\right)P_{1}}\sqrt{\gamma_{b}}\;\cdot erf(-\sqrt{\frac{1}{2\left[\frac{1}{\frac{3NM}{(M+1)P_{1}}\cdot \gamma_{b}}+\frac{1}{4{\left(\frac{3NM}{(M+1)P_{1}}\right)}^{2}{\gamma_{b}}^{2}}\right]}}$$

where $\gamma_{b}=\sum_{l=1}^{L}\lambda_{l}^{2}\frac{E_{b}}{N_{0}}$.

(27) $$\mu_{{\tilde{I}}_{m,j}}=\sqrt{\frac{2\sigma_{2}^{2}}{\pi }}=\sqrt{\frac{2[\frac{3NM}{(M+1){P_{1}}^{2}}+\frac{1}{2\gamma_{b}{P_{1}}^{2}}]}{\pi }}\cdot \sqrt{\sum_{l=1}^{L}\lambda_{l}^{2}\cdot E_{b}N_{0}}$$

Set $\sqrt{\frac{2[\frac{3NM}{(M+1){P_{1}}^{2}}+\frac{1}{2\gamma_{b}{P_{1}}^{2}}]}{\pi }}=\Gamma_{0}$; then the mean of ${\tilde{I}}_{m,j}$ can be expressed as

(28) $$\mu_{{\tilde{I}}_{m,j}}=\Gamma_{0}\cdot \sqrt{\sum_{l=1}^{L}\lambda_{l}^{2}\cdot E_{b}N_{0}}$$

The variance of $\left|I_{m,j}\right|$ is obtained as

(29) $$\sigma_{\left|I_{q,j}\right|}^{2}=\mu_{1}^{2}+\sigma_{1}^{2}-\mu_{\left|I_{q,j}\right|}^{2}=\sum_{l=1}^{L}\lambda_{l}^{2}\cdot E_{b}N_{0}\left[{\left(\frac{3NM}{\left(M+1\right)P_{1}}\right)}^{2}\gamma_{b}+\frac{3NM}{\left(M+1\right){P_{1}}^{2}}+\frac{1}{2\gamma_{b}{P_{1}}^{2}}-\Omega_{0}^{2}\right]$$

The variance of $\left|{\tilde{I}}_{m,j}\right|$ is obtained as

(30) $$\sigma_{\left|{\tilde{I}}_{m,j}\right|}^{2}=\mu_{2}^{2}+\sigma_{2}^{2}-\mu_{\left|{\tilde{I}}_{m,j}\right|}^{2}=\sigma_{2}^{2}-\mu_{\left|{\tilde{I}}_{m,j}\right|}^{2}=\sum_{l=1}^{L}\lambda_{l}^{2}\cdot E_{b}N_{0}\cdot \left(\frac{3NM}{\left(M+1\right){P_{1}}^{2}}+\frac{1}{2\gamma_{b}{P_{1}}^{2}}-\Gamma_{0}^{2}\right)$$

Based on Equation (8), the mean and the variance of $\xi_{q,j}$ can be respectively obtained as

(31) $$\mu_{\xi_{q,j}}=\mu_{\left|I_{q,j}\right|}-\mu_{\left|{\tilde{I}}_{q,j}\right|}=(\Omega_{0}-\Gamma_{0})\cdot \sqrt{\sum_{l=1}^{L}\lambda_{l}^{2}\cdot E_{b}N_{0}}$$

(32) $$\sigma_{\xi_{q,j}}^{2}=\sigma_{\left|I_{q,j}\right|}^{2}+\sigma_{\left|{\tilde{I}}_{q,j}\right|}^{2}=\sum_{l=1}^{L}\lambda_{l}^{2}\cdot E_{b}N_{0}\cdot \left[{\left(\frac{3NM}{\left(M+1\right)P_{1}}\right)}^{2}\gamma_{b}+\frac{6NM}{\left(M+1\right){P_{1}}^{2}}+\frac{1}{\gamma_{b}{P_{1}}^{2}}-\Omega_{0}^{2}-\Gamma_{0}^{2}\right]$$

Assuming constant bit energy and that the correlator output results from the subtraction of two folded Gaussian distributions, the decision metric produced by the subtractor can be modeled as a Gaussian random variable. The BER of the carrier index bits can be given as

(33) $$P_{eds}=\frac{1}{2}erfc\left(\frac{E\left[\xi_{q,j}\left|{\hat{s}}_{m,j}=1\right.\right]}{\sqrt{2Var\left[\xi_{q,j}\left|{\hat{s}}_{m,j}=1\right.\right]}}\right)$$

Substituting (31) and (32) into (33), the BER of the carrier index bits can be obtained as

(34) $$P_{eds}=\frac{1}{2}erfc\left\{{\left(\frac{{{(\Omega }_{0}-\Gamma_{0})}^{2}}{2\left[{\left(\frac{3NM}{\left(M+1\right)P_{1}}\right)}^{2}\gamma_{b}+\frac{3NM}{\left(M+1\right){P_{1}}^{2}}+\frac{1}{2\gamma_{b}{P_{1}}^{2}}-\Omega_{0}^{2}-\Gamma_{0}^{2}\right]}\right)}^{\frac{1}{2}}\right\}$$

#### 3.1.3. Derivation of $P_{edc}$

Based on Equation (7), considering q=m, the output observation variable of the $m^{th}$ branch is expressed as $\left|I_{m,j}\right|$.

However, when q≠m, the output observation variable of the $m^{th}$ branch is expressed as

(35) $$\left|I_{m,j}^{\prime }\right|=\left|\sum_{k=1}^{\theta }n_{m,k}\cdot \left(\sum_{k=1}^{\theta }\sum_{l=1}^{L}\lambda_{l}c_{x,k-\tau_{l}}+n_{R,k}\right)\right|$$

The mean and the variance of $I_{m,j}^{\prime }$ can be respectively obtained as

(36) $${E[I}_{m,j}^{\prime }]=0$$

(37) $$V_{ar}\left[I_{m,j}^{\prime }\right]=\frac{N_{0}^{2}}{4{P_{1}}^{2}}+\frac{N_{0}}{2P_{1}}\cdot \frac{3NME_{b}}{\left(M+1\right)P_{1}}\sum_{l=1}^{L}\lambda_{l}^{2}$$

Since the $2^{m_{c}}-1$ random variables $\left|I_{m,j}^{\prime }\right|$ are independent and their absolute values follow a folded normal distribution [31,32], the error code index detection probability $P_{edc}$ is computed by applying order statistics theory.

(38) $$P_{edc}=\int_{0}^{+\infty }(1-{\left[F_{\left|I_{m,j}^{\prime }\right|}\left(y\right)\right]}^{2^{m_{c}}-1})f_{\left|I_{m,j}\right|}(y)dy$$

where $F_{\left|I_{m,j}^{\prime }\right|}\left(y\right)$ is the cumulative distribution function (CDF) of $\left|I_{m,j}^{\prime }\right|$, given by

(39) $$F_{\left|I_{m,j}^{\prime }\right|}\left(y\right)=\frac{1}{2}\left[\mathrm{erf}\left(\frac{y-E[I_{m,j}^{\prime }]}{\sqrt{2V_{ar}\left[I_{m,j}^{\prime }\right]}}\right)+\mathrm{erf}\left(\frac{y+E[I_{m,j}^{\prime }]}{\sqrt{2V_{ar}\left[I_{m,j}^{\prime }\right]}}\right)\right]$$

$f_{\left|I_{m,j}\right|}(y)$ is the probability density function (PDF) of $\left|I_{m,j}\right|$, given as

(40) $$f_{\left|I_{m,j}\right|}\left(y\right)=\frac{1}{\sqrt{2\pi V_{ar}[I_{m,j}]}}\left[exp\left(-\frac{{\left(y-E{[I}_{m,j}]\right)}^{2}}{2V_{ar}\left[I_{m,j}\right]}\right)+\exp\left(-\frac{{\left(y+E{[I}_{m,j}]\right)}^{2}}{2V_{ar}\left[I_{m,j}\right]}\right)\right]$$

The error probability of the code index bits is expressed as

(41) $$P_{CIM}=\frac{2^{m_{c}-1}}{2^{m_{c}}-1}P_{edc}$$

where $P_{CIM}$ is a function of both the number of code index bits $m_{c}$ and the error code index detection probability $P_{edc}$ [32,33].

The BER of the carrier index bits can be calculated as

(42) $$P_{INDEX}=\left(1-P_{edc}\right)P_{eds}$$

#### 3.1.4. Derivation of $P_{MOD}$

To compute $P_{MOD}$, three error cases are considered. In the first case, both the code index bits and the carrier index bits are correctly detected, yet the MC-DCSK demodulation is erroneous. In the second case, correct code index detection occurs, while neither the carrier index bits detection nor the MC-DCSK demodulation is successful. In the last case, incorrect code index detection leads to a modulated bit demodulation error probability of 1/2.

Thus, the BER of the modulated bits $P_{MOD}$ can be obtained by

(43) $$P_{MOD}=\left(1-P_{edc}\right)\left(1-P_{eds}\right)P_{MC-DCSK}+\frac{1}{2}\left(1-P_{edc}\right)P_{eds}+\frac{1}{2}P_{edc}$$

where $P_{MC-DCSK}$ is the BER of the MC-DCSK.

Substituting $P_{CIM}$ (given in Equation (41)), $P_{INDEX}$ (given by Equation (42)) and $P_{MOD}$ (given by Equation (43)) into Equation (11), the BER of the proposed system can be derived.

In this analysis, independent and identically distributed Rayleigh slow fading channels are assumed. The probability density functions (PDFs) of the instantaneous SNR $\gamma_{a}$ is expressed as follows [34,35]

(44) $$f\left(\gamma_{b}\right)=\frac{\gamma_{a}^{L-1}}{{\left(L-1\right)!\bar{\gamma }}_{c}^{L}}\exp\left(-\frac{\gamma_{a}}{{\bar{\gamma }}_{c}}\right)$$

where the average bit SNR per channel is ${\bar{\gamma }}_{c}=\frac{E_{b}}{N_{0}}E[\lambda_{l}^{2}]$, and $\sum_{l=1}^{L}{E[\lambda }_{l}^{2}]=1$. Thus, the BER of the proposed system under multipath Rayleigh fading channel can be obtained by

(45) $$P_{system}=\int_{0}^{+\infty }\left(\frac{m_{c}}{m_{c}+m_{s}+m_{q}}P_{CIM}+\frac{m_{s}}{m_{c}+m_{s}+m_{q}}P_{INDEX}+\frac{m_{q}}{m_{c}+m_{s}+m_{q}}P_{MOD})f(\gamma_{a}\right)d\gamma_{a}$$

### 3.2. Efficiency Discussion

**Figure 3:**
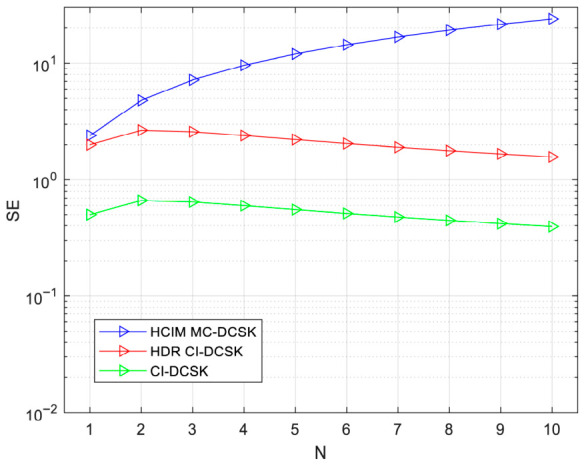
SE comparisons among HCIM MC-DCSK, HDR CI-DCSK, and CI-DCSK systems at different values of N. In each symbol period, the transmitted data consist of N carrier index bits, N code index bits, and N modulated bits, yielding a total of 3N bits per symbol.

This subsection compares the spectral efficiency (SE) and data rate of the proposed system with those of other systems, as summarized in Table 1. The SE comparisons are depicted in Figure 3. In our analysis, SE is defined as the number of bits transmitted per carrier in one symbol duration [36]. Each symbol carries N carrier index bits, N code index bits, and N modulated bits (3N bits in total). The data rate in the subsequent analysis is defined as the number of bits transmitted per unit time (bits/s) [37]. Evidently, the proposed HCIM MC-DCSK system demonstrates superior data rate performance compared to others. In Figure 3, we set M = 4. Based on the results in Figure 3, the proposed HCIM MC-DCSK system performs better than other systems in SE.

## 4. Simulation Results and Discussion

In this section, Monte Carlo simulations performed in MATLAB R2024a are conducted to confirm the theoretical analysis, running on a workstation with an Intel Core i9-13900K CPU and 64 GB RAM. The BER performance of the proposed HCIM MC-DCSK system is then compared with those of CI-DCSK and PT-CIM-DCSK systems to demonstrate the benefits of the proposed design.

Based on Equations (21) and (22), when the length of the Walsh codes increases, the variance will decrease. Then, the BER performance improves. Therefore, the BER performance improves as the length of the Walsh codes increases. The system complexity increases when the length of the Walsh codes increases. But the BER performance and the bit rate improve. Therefore, the trade-off between performance and system complexity can be selected according to actual needs. In this paper, we consider both not too much complexity and better system performance, so we set the length of the Walsh codes to 4 or 8.

**Figure 4:**
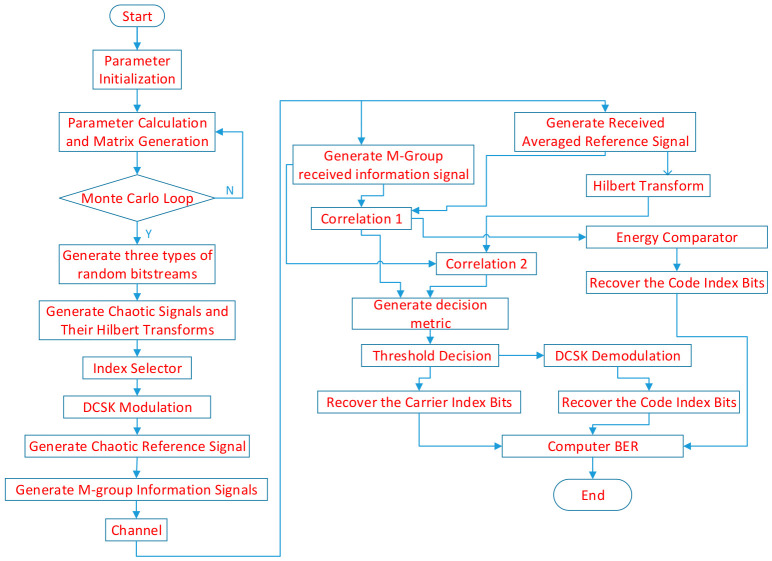
The flowchart of the simulation code.

The flowchart of the simulation code is shown in Figure 4.

**Figure 5:**
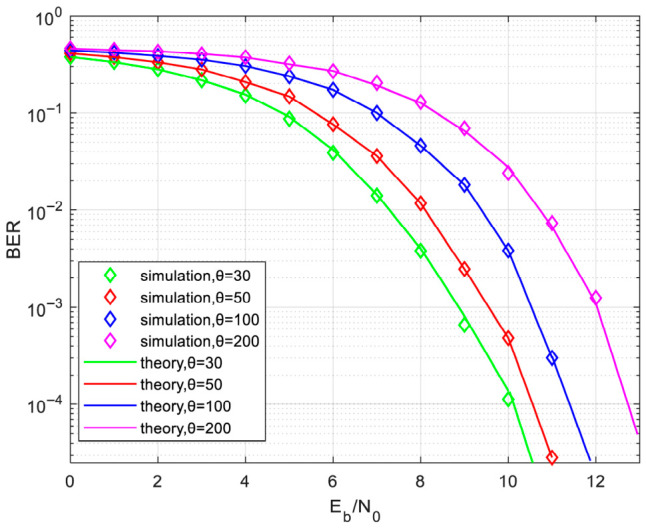
Theoretical and simulated BER results of the HCIM MC-DCSK system over an AWGN channel with different θ, N = 4, and M = 4.

The BER performance of the HCIM MC-DCSK system over an AWGN channel is illustrated in Figure 5, where theoretical curves are juxtaposed with simulation data. The strong concordance between these results validates the correctness of the theoretical analysis. Furthermore, for a fixed number of subcarriers, the BER performance of the HCIM MC-DCSK system degrades as θ increases from 30 to 200. As anticipated, higher values of θ result in poorer BER performance. This is attributable to the increased noise variance associated with larger spreading factors when the repeat times N and the length of the Walsh code $P_{1}$ are fixed.

**Figure 6:**
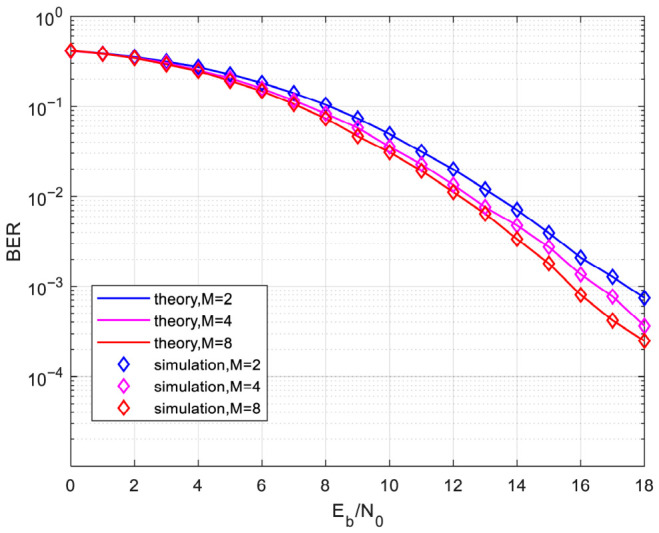
The BER performance comparison between theoretical derivations and Monte Carlo simulations over a multipath Rayleigh fading channel.

The BER performance comparison between theoretical derivations and Monte Carlo simulations over a multipath Rayleigh fading channel is depicted in Figure 6, where L=3, E(λ12)=E(λ22)=E(λ32)=13, and $\tau_{1}=0,\tau_{2}=2,\tau_{3}=5$. The simulation results confirm the theoretical accuracy under multipath Rayleigh fading conditions.

**Figure 7:**
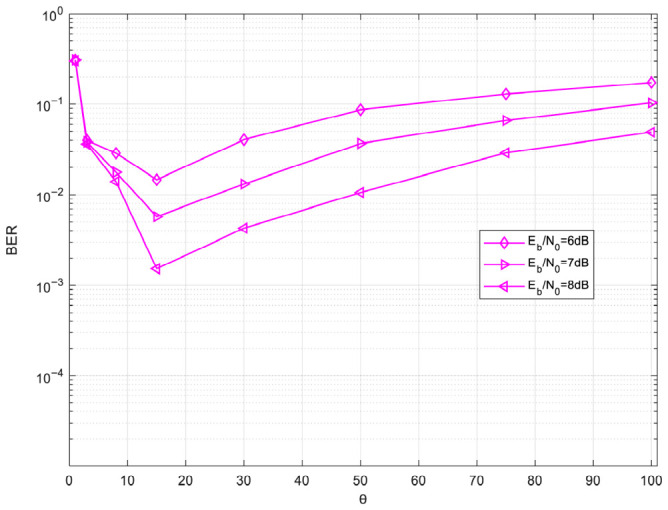
The dependence of HCIM MC-DCSK BER on the length of the chaotic signal (θ) in AWGN channels, with N = 4 and $\frac{E_{b}}{N_{0}}=6,7$ and 8 dB.

An analysis of the BER performance dependence on the length of the chaotic signal (θ) for the HCIM MC-DCSK system over AWGN is provided in Figure 7. At a given $E_{b}/N_{0}$, the BER demonstrates an initial decline with θ, followed by an optimal point, after which it increases once more. When the length of the chaotic signal increases, the noise component involved in the noise–noise correlation term becomes more significant, resulting in a poor BER performance. However, the complexity of the system will improve when the length of the chaotic signal increases. This interesting phenomenon strongly implies that HCIM MC-DCSK requires careful selection of an optimal θ. Furthermore, Figure 7 reveals that the optimal value of θ is approximately 15 in AWGN channels.

**Figure 8:**
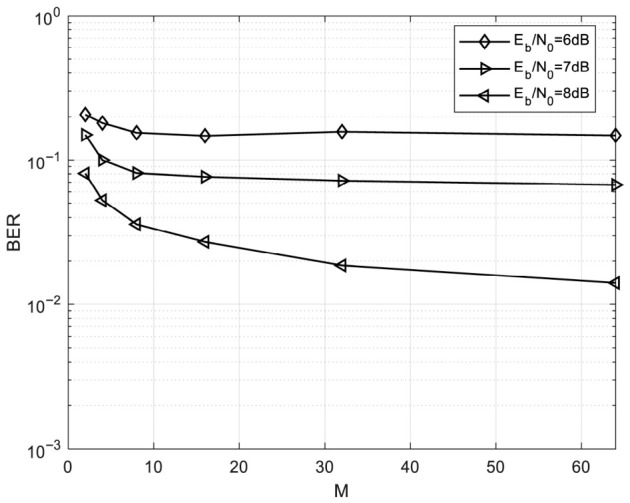
The dependence of HCIM MC-DCSK BER on the number of the subcarriers (M) in AWGN channels, with N = 4 and $E_{b}/N_{0}=$ 6, 7, and 8 dB.

An analysis of the BER performance dependence on the number of the subcarriers (M) for the HCIM MC-DCSK system over an AWGN channel is provided in Figure 8. Given fixed $E_{b}/N_{0}$ and N, the proposed system exhibits better BER performance as the number of subcarriers increases. For instance, increasing M from 2 to 64—while maintaining N=4 and $E_{b}/N_{0}=8$ dB—brings the BER of the proposed system down from 8 × 10^−2^ to 1.4 × 10^−2^. The proposed system exhibits rapid BER improvement as M increases from 2 to 16, followed by a gradual enhancement as M further increases from 16 to 64. Overall, higher subcarrier counts lead to better BER performance.

**Figure 9:**
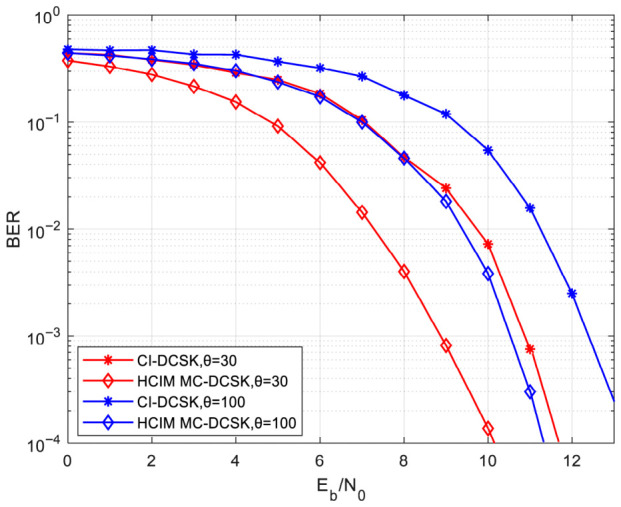
The comparison of the BER performance between CI-DCSK and the proposed HCIM MC-DCSK system over the AWGN channel.

Figure 8 illustrates the BER performance comparison between the CI-DCSK scheme [22] and the proposed HCIM MC-DCSK system. For the comparison in Figure 9, the system parameters are configured as follows: N=4 and M=4. Under identical parameter settings, the proposed system outperforms CI-DCSK in terms of BER performance. For instance, with θ set to 30, the proposed scheme exhibits a gain of approximately 2 dB at a BER level of $10^{-3}$ in the AWGN channel environment.

**Figure 10:**
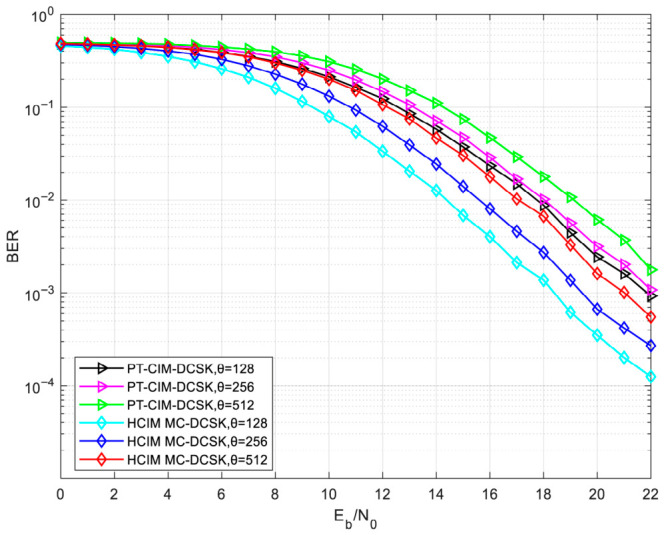
Comparison of BER performance between PT-CIM-DCSK and the proposed HCIM MC-DCSK system over a multipath Rayleigh fading channel.

The BER performance of the proposed HCIM MC-DCSK scheme is compared with that of PT-CIM-DCSK [38] over the multipath Rayleigh fading channel, as depicted in Figure 10. The comparative results in Figure 10 are obtained under the following conditions: length of Walsh codes $P_{1}=8$; number of data groups M=8. Simulation results indicate that, given the same parameters, the proposed system exhibits improved BER performance relative to PT-CIM-DCSK over the multipath Rayleigh fading channel. For instance, with θ set to 128, the proposed scheme exhibits a gain of approximately 4 dB at a BER level of $10^{-3}$ in the multipath Rayleigh fading channel environment.

**Figure 11:**
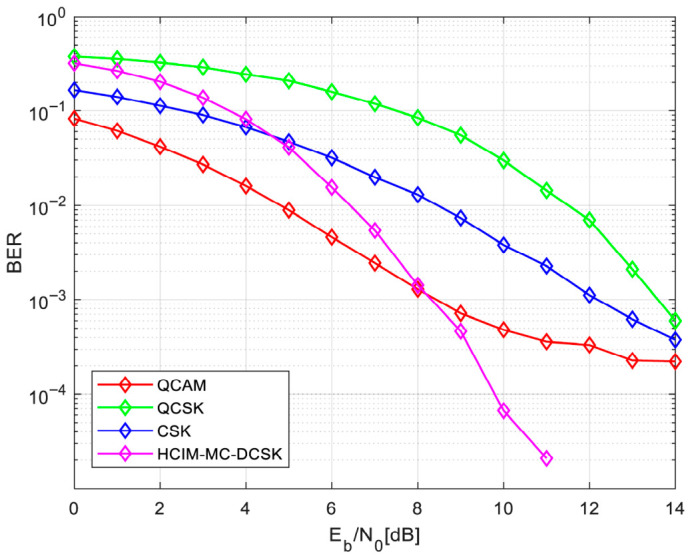
Comparison of BER performance between the proposed HCIM MC-DCSK system and three other systems over AWGN channel under the same length of the chaotic signal.

To place the proposed scheme in a broader context, in Table 2 and Figure 11 we compare it with systems such as CSK [3], QCSK [39], and QCAM [40,41]. In Table 2, the data rate, SE, and the number of channels of the proposed system and the other three systems are compared. Clearly, the proposed HCIM MC-DCSK system achieves a significantly higher data rate and SE than the other three systems. In each symbol period of the proposed system, the transmitted data consist of N carrier index bits, N code index bits, and N modulated bits, yielding a total of 3N bits per symbol. Figure 11 presents the BER performance comparison between the proposed system and the other three systems. Evidently, the BER performance of the proposed system is significantly better than that of the QCSK and CSK systems with the same length of the chaotic signal θ=15. Furthermore, the BER performance of the proposed system is better than that of QCAM when $E_{b}/N_{0}$ is greater than 8 dB.

## 5. Conclusions

In this paper, an HCIM MC-DCSK system is proposed. Theoretical BER expressions are formulated for the AWGN and multipath Rayleigh fading channels. The results demonstrate that decreasing the length of chaotic signal leads to better BER performance for HCIM MC-DCSK. Moreover, the BER performance of HCIM MC-DCSK will improve when the number of subcarriers increases. A performance comparison between HCIM MC-DCSK and CI-DCSK was conducted over the AWGN channel. The proposed scheme demonstrated superior BER performance compared to CI-DCSK when both systems operate with identical parameters. A performance comparison between HCIM MC-DCSK and PT-CIM-DCSK was conducted over the multipath Rayleigh fading channel. Simulation data show that the proposed system outperforms PT-CIM-DCSK in terms of BER under the same operating conditions. A performance comparison between HCIM MC-DCSK and three classic systems (CSK, QCSK, and QCAM) is also presented. The comparison results show that the data rate, SE and BER of the proposed system are better than those of three other systems. Thus, the proposed scheme offers better BER performance, increased data rate, and higher spectral efficiency concurrently.

## Figures and Tables

**Table 1:** Comparison of SE and data rate among different systems.

Performance	HCIM MC-DCSK	CI-DCSK	HDR CI-DCSK
SE	$\frac{M*3N}{M+1}$	$\frac{\log_{2}N+1}{N+1}$	$\frac{M(\log_{2}N+1)}{N+1}$
Data rate	M∗3N	$\log_{2}N+1$	$M(\log_{2}N+1)$

**Table 2:** Comparison between the proposed scheme and other systems.

Performance	HCIM MC-DCSK	QCSK	QCAM	CSK
SE	$\frac{M*3N}{M+1}$	2	4	1
Data rate	M∗3N	2	4	1
Number of channels	M+1	1	1	1

## Data Availability

The data supporting the findings of this study are available from the corresponding author upon reasonable request. Where applicable, restrictions due to privacy or ethical considerations apply.
